# Quantifying traditional Chinese medicine patterns using modern test theory: an example of functional constipation

**DOI:** 10.1186/s12906-016-1518-x

**Published:** 2017-01-13

**Authors:** Minxue Shen, Yuanwu Cui, Ming Hu, Linyong Xu

**Affiliations:** 1 Department of Dermatology, Xiangya Hospital, Central South University, Changsha, Hunan People’s Republic of China; 2 Hunan Key Laboratory of Skin Cancer and Psoriasis, Changsha, Hunan People’s Republic of China; 3 Department of Epidemiology and Health Statistics, Xiangya School of Public Health, Central South University, Changsha, Hunan People’s Republic of China; 4 The Second Affiliated Hospital of Tianjin University of Traditional Chinese Medicine, Tianjin, People’s Republic of China

**Keywords:** Multidimensional item response theory, Scale validation, Traditional Chinese medicine, Functional constipation

## Abstract

**Background:**

The study aimed to validate a scale to assess the severity of “*Yin* deficiency, intestine heat” pattern of functional constipation based on the modern test theory.

**Methods:**

Pooled longitudinal data of 237 patients with “*Yin* deficiency, intestine heat” pattern of constipation from a prospective cohort study were used to validate the scale. Exploratory factor analysis was used to examine the common factors of items. A multidimensional item response model was used to assess the scale with the presence of multidimensionality.

**Results:**

The Cronbach’s alpha ranged from 0.79 to 0.89, and the split-half reliability ranged from 0.67 to 0.79 at different measurements. Exploratory factor analysis identified two common factors, and all items had cross factor loadings. Bidimensional model had better goodness of fit than the unidimensional model. Multidimensional item response model showed that the all items had moderate to high discrimination parameters. Parameters indicated that the first latent trait signified intestine heat, while the second trait characterized *Yin* deficiency. Information function showed that items demonstrated highest discrimination power among patients with moderate to high level of disease severity.

**Conclusions:**

Multidimensional item response theory provides a useful and rational approach in validating scales for assessing the severity of patterns in traditional Chinese medicine.

**Electronic supplementary material:**

The online version of this article (doi:10.1186/s12906-016-1518-x) contains supplementary material, which is available to authorized users.

## Background

Traditional Chinese medicine (TCM) is an important complimentary and alternative medicine approach that is widely used in China, and it is becoming prevalent in industrialized countries [1]. TCM treats the biological body as a microcosm of the basic natural forces at work in the universe. It seeks the underlying etiological mechanisms based on the prestigious *Yin*-*Yang* and five phase (*Wu Xing*) theory [2]. In TCM, a disease has two aspects: disease entity and pattern. Pattern is more important because it explains the etiology of a disease entity, and the therapy will be chosen according to the pattern rather than disease: patients with the same disease entity but different patterns will receive different therapy; vice versa, patients with similar patterns may receive similar therapy even if their diseases or clinical manifestations are different [3]. The disharmony patterns, or etiologic mechanisms of diseases, are described as combinations of affected body elements in TCM (i.e. *Qi*, blood, body fluids, organs, and meridians).

The most important step of pattern diagnosis (differentiation) is evaluation of the signs and symptoms. However, because scientific investigation has found no histological evidence for TCM concepts including *Qi* and meridians, the validity of pattern diagnosis has been constantly suspected [4]. Since patterns are not directly observable and the severity is not measurable, latent variable model might be useful in this scenario [5]. In contrast to classical test theory, modern test theory assesses the adequacy of a measure using an item-based approach that specifies a nonlinear relationship between responses (presence or severity of symptoms and signs) and the latent trait (the TCM pattern) [6]. It provides item-specific information of a test and avoids weight bias owing to subjective allocation of weight to each item [7].

**Fig. 1:**
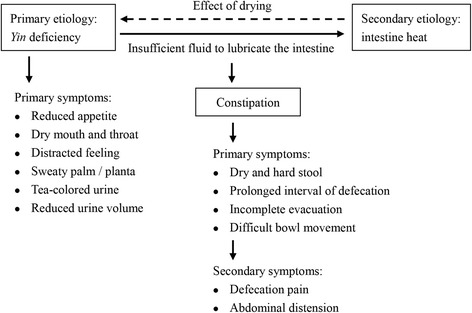
Mechanism of constipation caused by *Yin* deficiency in Chinese medicine

In current study, we used the “*Yin* deficiency, intestine heat” pattern of constipation as an example to illustrate the effectiveness of this modern approach in quantifying the severity of TCM pattern. Constipation is a common gastrointestinal complaint clinically, affecting an estimated 12–19% of Americans, 14% of Asians and up to 27% of the global population [8, 9], and significantly impacts on health-related quality of life [10]. With an unfavorable response to current treatments, many patients in China seek help from TCM, mostly taking herbal medicine [9]. In TCM, constipation is divided into excessive and deficient patterns in general [2]. The former is characterized by presence of heat or *Qi* stagnation, and the latter is characterized by depletion of *Qi*, *Yin* or *Yang*. We recruited patients with the pattern of *Yin* deficiency intestine heat. *Yin* deficiency leads to insufficient fluid to lubricate the intestine, and heat results in constipation by drying the intestine and stool. Patients present with hard, dry and pellet-like stools, difficulty in passing stools, reduced appetite, tea-colored urine, red complexion, dry mouth and throat, sweaty palm and planta, red tongue with thin coat, and thready and rapid pulse (Fig. 1).

Previous studies involving Chinese herbal medicines used the 7-point Bristol Stool Scale [11] and Wexner Constipation Scale [12] to evaluate the severity of constipation and the efficacy of treatment, regardless of TCM patterns [13, 14]. However, it has been reported that those scales cannot distinguish different patterns of constipation well [15]. In our study, we validated a pattern-specific tool that might be useful in estimating the efficacy of a therapy in relieving the external manifestations as well as correcting its underlying pathologic imbalances.

## Methods

### Data source

Prospective cohort study design was used. Patients aged 18–70 from four TCM hospitals in Hunan, Jiangxi, Henan, and Guangxi province were assessed by the scale in 2009. Constipated patients were diagnosed according to Rome III criteria, i.e. at least two of the following occurrences for more than 25% of the time: straining, lumpy or hard stools, sensation of incomplete evacuation, sensation of anorectal obstruction or blockage, use of manual maneuvers to facilitate, or less than three defecation per week [16].

Patients with pattern of *Yin* deficiency intestine heat were diagnosed based on the presence of at least one symptom in each of the following categories: (1) dry and hard stool; prolonged interval of spontaneous defecation; (2) a feeling of incompleteness; defecation pain; difficult bowel movement; abdominal distension; reduced appetite; (3) dry mouth and throat; sweaty palm and planta, and distracted feeling; tea-colored urine, and reduced urine volume; (4) red tongue with thin coat; thready and rapid pulse.

Exclusion criteria were a history of organic gastrointestinal diseases such as colorectal cancer, advanced colonic polyps, enterophthisis, or inflammatory bowel disease; systematic diseases that might cause constipation; abdominal surgery; severe cardiologic, neurologic, hepatic, endocrine, metabolic, hemopoietic, or psychiatric diseases; allergies; pregnant or breast feeding women; those who used constipation medications in past four weeks; positive stool occult blood.

### Tool and measurement

A scale was developed by TCM experts to evaluate the severity of *Yin* deficiency intestine heat pattern of constipation. The scale included ten items; there were four categories within each item (Table 1). The items characterized the features of constipation under an etiology of *Yin* deficiency intestine heat. Patients were assessed using this scale before treatment, at 7th day after treatment (1st follow-up), and at 14th day after treatment (2nd follow-up), respectively. Patients were evaluated by their doctors through interviews, so that the response style effects in patient reported outcomes could be minimized [17]. Response style effects refers to the phenomenon of content-irrelevant or nuisance factors (such as personality traits) systematically influencing and distorting responses to survey questions. The scale was assessed using a mixture of methods including modern test theory and classical test theory.

**Table 1:** Scale to assess the *Yin* deficiency intestine heat pattern of constipation

	Definitions of item categories			
	0	1	2	3
1. Dry and hard stool	Negative	Slightly	Moderately	Extremely
2. Prolonged interval of spontaneous defecation	Negative	Interval ≥ 72 h	Interval ≥ 96 h	Interval ≥ 120 h
3. Sensation of incomplete evacuation	Negative	Slightly; occasionally	Moderately; sometimes	Severely; usually
4. Defecation pain	Negative	Slightly; occasionally	Moderately; sometimes; no obvious impact on defecation	Severely; always; afraid of defecation because of pain
5. Difficult bowel movement	Negative	Slightly	Moderately; arduously	Extremely difficult; need assistance
6. Abdominal distension	Negative	Slightly	Moderately; tolerable	Severely; intolerable
7. Reduced appetite	Negative	Slightly	Moderately; reduced food intake ≥ 1/3	Severely; reduced food intake ≥ 1/2
8. Dry mouth and throat	Negative	Slightly; occasionally	Moderately; sometimes	Severely; usually; increased water intake
9. Sweaty palm and planta; distracted feeling	Negative	Slightly; occasionally	Moderately; sometimes	Severely; feel distracted all the day
10. Tea-colored urine, reduced urine volume	Negative	Slightly	Moderately tea-colored; reduced urine volume ≥ 1/4	Extremely tea-colored; reduced urine volume ≥ 1/3

### Statistical methods

Classical test theory was used to assessed the reliability of the scale. Cronbach’s alpha and Spearman-Brown split-half reliability were estimated using the baseline as well as follow-up data. Test-retest reliability was not estimated because patients had received treatment at the 1st follow-up. Pearson’s correlation coefficients between item score and summed score were estimated. Exploratory factor analysis (EFA) was used to determine number of factors. Factors with eigenvalue ≥ 1.0 were remained. Goodness of model fit (unidimensional vs. bidimensional model) were compared according to log-likelihood, Akaike information criterion, and Bayesian information criterion.

Because most items had cross factor loadings, multidimensional item response theory (MIRT) analysis was used to assess the psychometric properties of the items and the reliability of the scale. Item response theory (IRT) is a family of associated mathematical models that relate latent traits (or ability) to the probability of endorsing items in an assessment. It describes a nonlinear relationship between binary, ordinal, or categorical responses and the latent trait. When the response to an item is associated with more than one ability, the unidimensionality hypothesis of IRT is compromised. In order to solve the ubiquitous multidimensionality issue of a measure, Mulaik proposed the MIRT for dichotomous items. Later, Muraki and Carlson proposed the multidimensional grade response model (MGRM) in the form of cumulative normal distribution function. In current study, we applied a compensatory logistic MGRM to simplify the estimation of parameters. In equation (1), *P*_*ijk*_ refers to the probability of subject *j* responding to category *k* (and above) of item *i*; ***a***_***i***_ is the discrimination parameter vector of item *i*; ***θ***_***j***_ is the ability vector of subject *j*; *d*_*ik*_ is the easiness parameter of category *k* of item *i*; E(***θ***_***j***_) is the expected score (the linear accumulation of probability of responding to each category of an item) of subject *j* with ability vector ***θ***_***j***_. It is worth noting that the easiness parameter *d*_*ik*_ is similar to the difficulty parameter in unidimensional IRT, whereas their symbols are opposite. The guessing parameter was not considered in our study. Parameters were estimated by the Markov chain Monte Carlo (MCMC) method. A maximum of 4,000 cycles was allowed in MCMC estimation.

1 $$ {P}_{ijk}=\frac{ \exp \left({\boldsymbol{a}}_{\boldsymbol{i}}{\boldsymbol{\theta}}_{\boldsymbol{j}}+{d}_{ik}\right)}{1+ \exp \left({\boldsymbol{a}}_{\boldsymbol{i}}{\boldsymbol{\theta}}_{\boldsymbol{j}}+{d}_{ik}\right)}\begin{array}{cc}\hfill, \hfill & \hfill E\left({\boldsymbol{\theta}}_{\boldsymbol{j}}\right)={\displaystyle \sum_{k=1}^{k_i}{P}_{ijk}}\hfill \end{array} $$

Pooled baseline and follow-up panel data were used to estimate the MIRT parameters. A mixture of panel data will increase the variation of ability (i.e. severity of pattern), and a more reliable and stable estimation of IRT parameters will be obtained. Although repeated measurements are dependent within the subject, the “local dependence hypothesis” of IRT is not compromised because the ability per se at any measurement reflects its actual status at that time point, and it is not likely to be impacted by doctors or other patients.

Item information describes the precision of an item. An item is most useful among participants with ability vector corresponding to the peak of information surface. For logistic MGRM, the item function can be estimated as the following [18]. In equation (2), the definitions of *P*_*ijk*_, ***a***_***i***_, and ***θ***_***j***_ are identical to those in equation (1); ***α***_***v***_ is the direction vector, and *I*_*iα*_ is item information function. Because information surface changes with the direction of observation, we set *α* to 45° for all items in our study.

2 $$ {I}_{i\alpha}\left({\boldsymbol{\theta}}_{\boldsymbol{j}}\right)={\displaystyle \sum_{k=0}^{k_i}\left({P}_{ij,k}-{P}_{ij,k+1}\right)}{\left(1-{P}_{ij,k}-{P}_{ij,k+1}\right)}^2\Big({\displaystyle \sum_{v=1}^m{\boldsymbol{a}}_{\boldsymbol{iv}} \cos {\boldsymbol{\alpha}}_{\boldsymbol{v}}\Big){}^2} $$

MIRT analysis was performed in IRTPRO 3.1 (Scientific Software International Inc., Lincolnwood). Expected score surfaces and item information surfaces were visualized using MATLAB 7.0 (MathWorks Inc., Natick, Massachusetts). Other statistical analyses were performed using SAS 9.4 (SAS Institute Inc., Cary, North Carolina). Significance level was 0.05 for all statistical tests.

## Results

A total 239 patients were diagnosed as constipation by both Rome III criteria and TCM criteria. Two patients were excluded: one was younger than 18; the other rejected to participate in the project. 237 patients were included in the statistical analysis for scale validation. Two patients were lost at the 2nd follow-up. The demographic information of the patients is shown in Table 2. The Cronbach’s alpha ranged from 0.79 to 0.89, and the split-half reliability ranged from 0.67 to 0.79 at different measurements (Table 3).

**Table 2:** Demographic characteristics of patients

		*N* (%)
Age (years)	19–29	77 (32.5)
	30–39	45 (19.0)
	40–49	42 (17.7)
	50–59	51 (21.5)
	≥60	22 (9.3)
Sex	Male	67 (28.3)
	Female	170 (71.7)
Marriage	Married	163 (68.8)
	Unmarried	74 (31.2)
Ethnics	Han	232 (97.9)
	Other	5 (2.1)

**Table 3:** Reliability of the scale

	Baseline	1st follow-up	2nd follow-up
Cronbach’s alpha	0.78	0.87	0.89
Split half	0.67	0.77	0.79

EFA showed that two factor have eigenvalue > 1. All indices presented in Table 4 suggested that the bidimensional model provided a better fit. The Pearson’s correlation coefficients between item score and summed score are shown in Table 5. All items had cross factor loadings on the two factors. Most items had at least moderate loading (*λ* ≥0.4) on factor 2, while item 7–10 had low loadings (*λ* <0.4) on factor 1. Item 1–5 had higher loadings on factor 1, while item 7–10 had significantly higher loadings on factor 2.

**Table 4:** Comparison of goodness of model fit

	Unidimensional model	Bidimensional model
–2 log-likelihood	12632.2	12405.6
Akaike information criterion	12712.2	12505.6
Bayesian information criterion	12894.8	12733.8

**Table 5:** Estimation of factor loadings and MIRT parameters of items using the pooled panel data

	Pearson’s correlation	Factor loadings		MIRT parameters				
		*λ*_1_ (SE)	*λ*_2_ (SE)	*a*_1_ (SE)	*a*_2_ (SE)	*d*_1_ (SE)	*d*_2_ (SE)	*d*_3_ (SE)
1. Dry and hard stool	0.76*	0.76 (0.07)*	0.70 (0.07)*	2.67 (0.31)*	1.54 (0.23)*	3.07 (0.30)*	−1.56 (0.20)*	−5.96 (0.45)*
2. Prolonged interval of spontaneous defecation	0.73*	0.70 (0.07)*	0.48 (0.09)*	2.20 (0.24)*	1.50 (0.20)*	1.70 (0.20)*	−3.09 (0.23)*	−6.36 (0.46)*
3. Sensation of incomplete evacuation	0.65*	0.70 (0.06)*	0.32 (0.11)^#^	1.85 (0.17)*	0.86 (0.18)*	2.60 (0.20)*	−1.24 (0.14)*	−5.56 (0.40)*
4. Defecation pain	0.78*	0.64 (0.08)*	0.53 (0.10)*	1.97 (0.20)*	1.62 (0.21)*	1.75 (0.18)*	−1.53 (0.17)*	−5.57 (0.32)*
5. Difficult bowel movement	0.82*	0.68 (0.08)*	0.56 (0.10)*	2.46 (0.26)*	2.02 (0.25)*	3.31 (0.27)*	−0.67 (0.19)*	−5.26 (0.38)*
6. Abdominal distension	0.77*	0.54 (0.08)*	0.60 (0.08)*	1.57 (0.16)*	1.73 (0.18)*	2.13 (0.18)*	−1.36 (0.15)*	−6.12 (0.38)*
7. Reduced appetite	0.73*	0.43 (0.09)*	0.65 (0.08)*	1.17 (0.16)*	1.79 (0.19)*	0.31 (0.13)*	−2.51 (0.18)*	−5.52 (0.35)*
8. Dry mouth and throat	0.73*	0.25 (0.09)^#^	0.79 (0.05)*	0.78 (0.17)*	2.45 (0.22)*	1.44 (0.17)*	−2.07 (0.20)*	−6.06 (0.46)*
9. Sweaty palm and planta; distracted feeling	0.69*	0.13 (0.13)	0.88 (0.06)*	0.50 (0.25)^#^	3.35 (0.52)*	0.16 (0.20)*	−3.91 (0.51)*	−8.04 (0.98)*
10. Tea-colored urine, reduced urine volume	0.64*	0.22 (0.10)^#^	0.71 (0.06)*	0.57 (0.16)*	1.82 (0.18)*	0.62 (0.13)*	−2.75 (0.20)*	−6.70 (0.55)*

*SE* standard error. *λ*_*i*_: factor *i. a*_*i*_: discrimination parameter of dimension *i. d*_*j*_: easiness parameter of category *j* of an item

^#^ *P* <0.05. *P <0.01

**Fig. 2:**
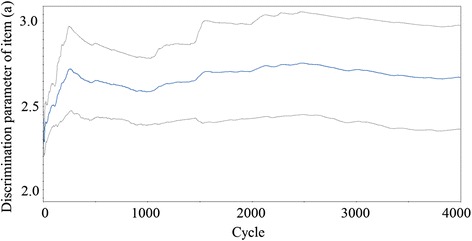
An example of MCMC estimation for MIRT parameters. The mean of discrimination parameter *a*_*1*_ of item 1 converged at 2.67 after 4,000 cycles of simulation

MIRT parameters were estimated using the MCMC method. An example of MCMC process is presented in Fig. 2. The mean of discrimination parameter *a*_*1*_ of item 1 converged at 2.67 after 4,000 cycles of simulation. MIRT analysis showed that all items have acceptable discrimination parameters (*a*_*i*_ ≥0.5). Many items had high (*a*_*i*_ ≥1.5) and very high (*a*_*i*_ ≥2.0) discriminative power (Table 5). Consistent with factor analysis, item 1–5 had significantly higher discrimination on the first trait, while item 7–10 had higher discrimination on the second trait.

**Fig. 3:**
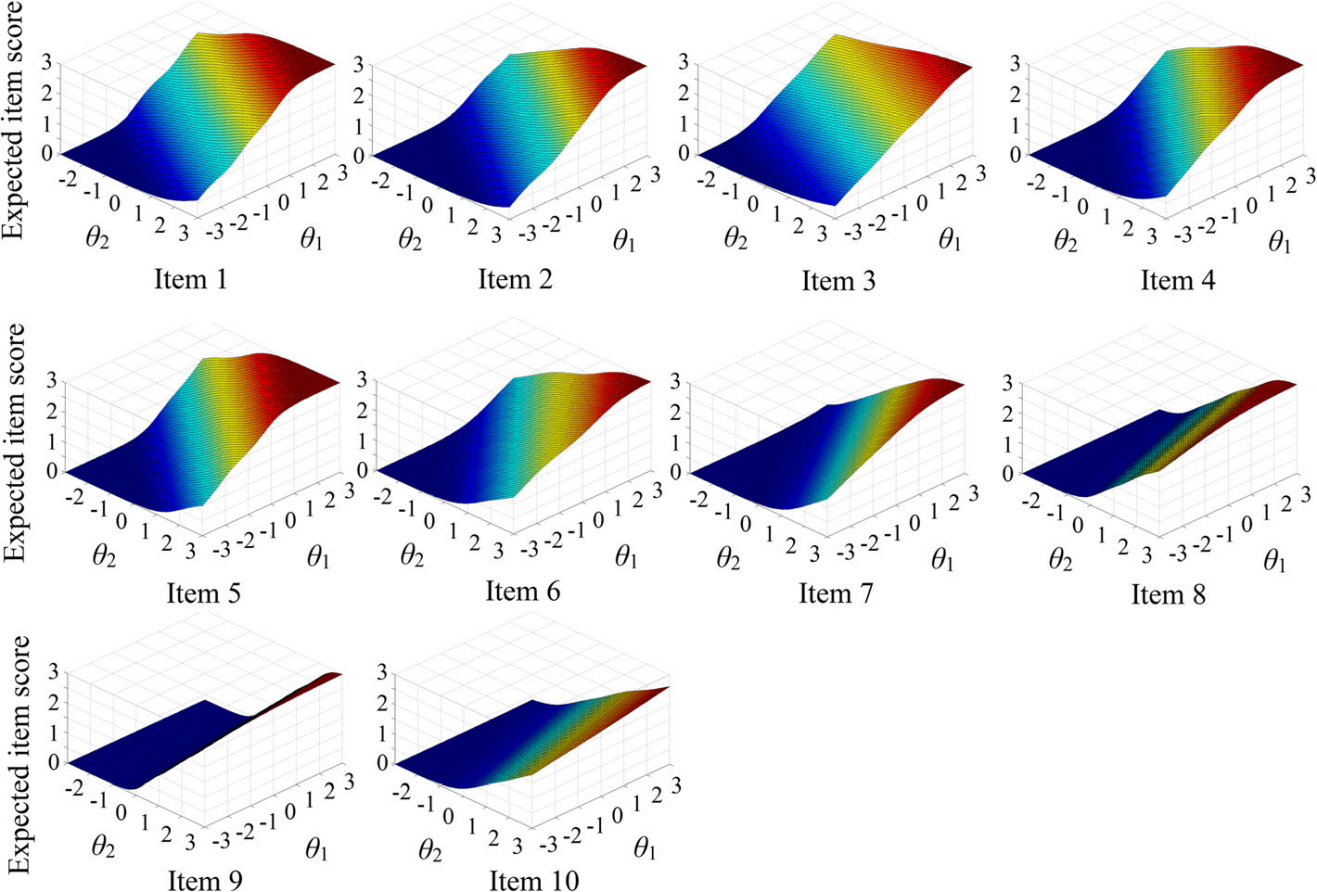
Expected item score surfaces. Each panel represents the expected score surface of an item, with corresponding item number below the panel. X- and Y-axis characterize the level of two latent traits respectively. Z-axis refers to the expected item score, i.e. the linear accumulation of probability of responding to each category of an item. Expected score reaches its peak when both latent traits were approximate to 3.0 (maximum). Latent trait 1 has greater impacts on responses to item 1–5, while latent trait 2 has greater impacts on responses to item 7–10. Item six has an approximately symmetric surface (the two latent traits have similar impacts on the item response)

Expected score surface signifies the non-linear relationships between latent traits and accumulated probability of endorsing different categories of an item. Figure 3 shows that the first trait had greater impacts on responses to item 1–5; it had significant compensatory effects for the second trait. For item six, the surface was approximately symmetric; i.e., the two traits had similar impacts on item response. For item 7–10, the second trait had greater impacts on the expected scores; it had overwhelming compensatory effects for the first trait.

**Fig. 4:**
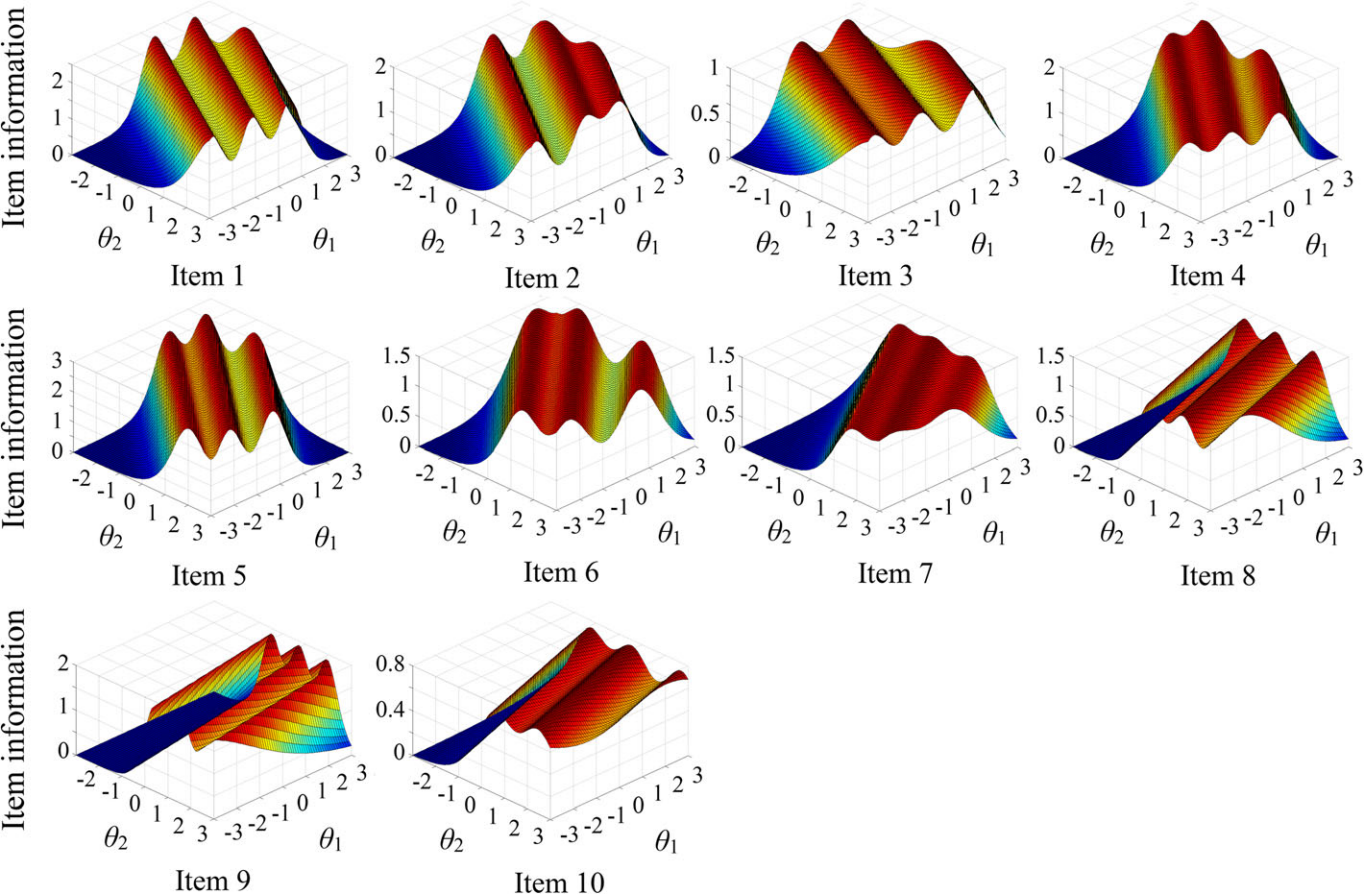
Item information function surfaces. Each panel represents the information function surface of an item, with corresponding item number below the panel. X- and Y-axis characterize the level of two latent traits respectively. Z-axis refers to the item information (observation direction = 45°). An item is most useful when the item information peaks. Owing to the categorical nature of the responses, all item information function surfaces have multiple peaks. Generally, items provide most information among patients with moderate severity (ability between –1 and 2). When both latent traits are close to –3 or 3, the information is approaching to zero

Item information function surfaces are shown in Fig. 4. Owing to the categorical nature of item responses, the surfaces had multiple peaks. Generally, items had maximum information among patients with latent trait levels between –1 and 2. When the abilities of both dimensions were close to –3 (least severe) or 3 (most severe), the information was approaching to zero. The information surfaces indicated that the items were most discriminative among patients with moderate to high severity of the pattern, but were becoming useless among those with minimum or maximum severity.

## Discussion

Our study validated a scale to evaluate the severity of the “*Yin* deficiency, intestine heat” pattern of constipation using the pooled data of longitudinal measurements among 237 patients in a prospective cohort study. Overall, classical test theory showed good Cronbach’s alpha coefficients and Spearman-Brown split-half reliability. Test-retest reliability was not estimated owing to the TCM treatment. Two factors were identified in EFA, and all items had cross factor loadings with different magnitude. MIRT showed that both latent traits were associated with the responses to items. The first trait was associated with responses to items 1–5 with greater magnitude, while the second trait was generally associated with responses to all items. It could be interpreted that the first latent trait signified intestine heat that was more associated with the constipation symptoms, while the second latent trait signified *Yin* deficiency which was characterized by symptoms (reduced appetite, dry mouth and throat, sweaty palm and planta, distracted feeling, tea-colored urine, and reduced urine volume) more than constipation. Overall, the scale showed good psychometric properties. The items provided most information among patients with moderate to high ability levels (i.e. severity of the pattern). The MIRT parameters could be well explained by the TCM theory.

In classical test theory, assessment of reliability is inaccurate and obscure. Cronbach’s alpha assesses the overall consistency of a scale, but item-level measurement errors are not specified. Split-half reliability and test-retest reliability are proposed under the hypothesis of “parallel test”. However, a real parallel test is not possible in practice.

In EFA, two factors were identified and all items had cross factor loadings on them. The bidimensional model had better fit to the response data than the unidimensional model. As a result, IRT model is not suitable for our data because the unidimensionality assumption is compromised. The scale is a measure of two distinct but mutually correlated latent traits. Because *Yin* deficiency results in intestine heat at first, and the latter exacerbates the former in turn, we used a compensatory model in this study. The compensatory MIRT allows dimensions to combine linearly to produce probability of endorsing an item; that is, high ability in a dimension can compensate lower ability in other dimensions. Many items had very high discrimination parameters (*a*_*i*_ ≥ 2.0). The item information function surfaces showed that these items were most discriminative among patients with moderate to high severity of the pattern. The high discrimination parameter as well as the peaked information function indicated the quasi-traits of a TCM construct. Quasi-trait refers to a unipolar construct in which one end of the scale represents a disease, while the other pole represents its absence [19]. This is in contrast to a bipolar construct (such as literacy and knowledge) where both ends of the scale represent meaningful variation [20, 21]. In clinical settings, a construct of quasi-trait(s) is more useful in assessing the severity of a disease, because the minimum ability level indicates the absence of a disease or pattern rather than health.

Apart from good test properties of the scale, the MIRT results could also be well explained by the TCM theory. According to TCM, depletion of *Yin* results in insufficient fluid to nurture the body tissues including the intestine. As a result, *Yin* deficiency is the primary etiology of constipation of this pattern, and it has overall impact on all items with different magnitude. In contrast, intestine heat is the downstream of *Yin* deficiency and is more associated with the constipation symptoms. This explains why the second trait had moderate to high factor loadings and discrimination parameters on all items, while the first trait only had high loadings on item 1–5.

The study has several limitations. First, although MIRT provides a potentially useful approach for better understanding and generalizing TCM with respect to pattern diagnosis and assessment of effectiveness, the technique does not actually solve the pseudoscience controversy towards TCM. Second, the study recruited constipated patients only, and the severity of disease pattern lacked enough variation, although by pooling the longitudinal data (Additional file 1), the variation was increased. Generally, sample size ≥ 500 will suffice accurate MIRT parameter estimates [22]. Negative controls should also be included in the validation of the tool. Third, the study population was consist of Chinese adults, and might not be generalizable to other populations. Last, because the item information function has a direction vector, the maximum information of an item depends on the observation direction chosen by investigator. How to maximize the information provided by the items still remains an unsolved problem in MIRT research.

In spite of the limitations, the study has strengths. To our knowledge, this is the first study to validate a TCM scale based on the modern test theory. MIRT provides a rational model to fit the data and the results can be well explained by the TCM theory. Although multidimensionality is ubiquitous in medical research [23], many studies ignored this issue. Second, the data is derived from a prospective cohort study, and the pattern diagnosis of constipation and the quality of the data were under meticulous control. Third, because the scale items were semi-quantitative and patients were assessed by physician through interviews, response style effects in patient reported outcomes were less likely to occur [24].

## Conclusions

A brief scale to assess the severity of "*Yin* deficiency, intestine heat" pattern of functional constipation was validated based on a multidimensional item response model. The scale was characterized by bidimensional structure, and demonstrated good discrimination power. Multidimensional item response theory provides a useful and rational approach to quantifing traditional Chinese medicine.

## Additional files

Additional file 1: S1. Longitudinal data used for the validation of the tool. With respect to variable names, T1, T2, and T3 prefix indicate baseline, 1st follow-up, and 2nd follow-up, respectively. I1 to I10 suffix indicate number of item. (XLSX 32 kb)
